# A Case of Early Menopause

**Published:** 1906-06

**Authors:** Emory Lanphear

**Affiliations:** St. Louis, Mo.


For Texas Medical Journal.

### A Case of Early Menopause.

BY EMORY LANPHEAR, M. D., PH. D., LL. D., ST. LOUIS, MO.

Early appearance of the menopause is quite frequent, i. e., "change of life" appearing at 40, 38 or even 35, in rare instances. But that it should come at 28 is certainly astonishing. Such a case, however, appears to be the following:

Mrs. H. E., patient of Dr. Geo. S. Dodd, of Brookport, Ill.; was quite "sickly" during early childhood and slow in maturing. First menstruation appeared at 15, but was not fully established until 18. She was married at 23 and had no gonorrhea following it—so far as can be determined by the history obtainable—certainly no pelvic peritonitis of sufficient violence to confine her to bed, as would be expected when the fimbriated extremity of the Fallopian tubes is obliterated by gonorrheal salpingitis — a most prolific source of sterility, by the way, in country as well as city. (So many young fellows get gonorrhea when they take a car of hogs to the city!) She never became pregnant. When about 25 years of age she had tuberculosis of the knee ("white swelling"), and for a time it was thought she would die of consumption. At 28 the menses stopped, with considerable disturbance: headaches, flushings, periodical backaches, etc., and they have never since reappeared.

Examination shows the patient to be in most excellent physical condition. The uterus is about three inches long, slightly anteverted and freely movable. The cervix is elongated slightly but freely patulous. So far as can be determined there is no occlusion of the tubes. The ovaries can not be felt—so far presumed to be normal in position and size, i. e., certainly not greatly enlarged, though they may be atrophied.

She is slightly older in her looks than her years—as would be expected in a woman ten years past the menopause.

I have no explanation of its early appearance.

---

GUADALUPE STANDS PAT.

WHEREAS, The class of examinations required by life insurance companies is of a nature which entails responsibility and which, in justice to the medical profession, should not be made for less than the fee of $5, heretofore allowed, and whereas, we charge a similar fee to private patients for examinations of like character; therefore, be it

*Resolved,* That we, the undersigned members of the Guadalupe County Medical Society, hereby agree to make no examinations for any life insurance company or society for a less fee than $5.

*Resolved further,* That these resolutions be forwarded to the respective life insurance companies whose accredited examiners we are.

M. B. GRACE,
R. L. KNOLLE,
O. G. PEARSON,
W. MYERS,
T. W. MOORE,
A. M. STAMPS,
S. S. BEAKLY,
Seguin, Texas.
J. M. EHLERT,
Kingsbury, Texas.
J. W. WILLIAMS,
Staples, Texas.
LOUIS HIRSCHFELD,
E. G. BURGES,
Marion, Texas.
D. A. WATSON,
Schertz, Texas.

